# Importance of age and timing of referral when initiating dialysis

**DOI:** 10.1007/s40620-025-02390-7

**Published:** 2025-08-26

**Authors:** David Antoine Jaques, Anne Dufey, Cyrielle Alves, Sophie De Seigneux, Patrick Saudan

**Affiliations:** Division of Nephrology, Geneva University Hospitals, Rue Gabrielle-Perret-Gentil 4, 1205 Geneva, Switzerland

**Keywords:** Dialysis, Elderly, Late referral, Mortality

## Abstract

**Background:**

Mortality of patients > 75 years of age initiating dialysis is high. Late referral to a nephrologist prior to dialysis initiation is associated with poor outcomes. Herein, we report the outcomes of patients initiating dialysis according to their age and timing of referral.

**Methods:**

We reviewed a prospective cohort of patients initiating dialysis from 2000 to 2022 at a single university center. Primary outcome was one-year all-cause mortality. Secondary outcomes were overall all-cause mortality and one-year hospitalization days. Late referral was defined as dialysis initiation < 1 month after a first consultation with a nephrologist.

**Results:**

We included 906 patients, including 246 (27%) aged over  75 years. Late referral was more common in elderly patients compared to younger ones, with rates of 26% and 34%, respectively (*p* = 0.027). Regarding one-year mortality, considering patients aged over 75 years with early referral as the reference, patients aged > 75 years with late referral were at higher risk (Hazard Ratio [HR] 2.30, *p* = 0.001), while patients aged < 75 years with either early or late referral were at similar risk. Regarding overall mortality, patients aged > 75 years with late referral were at higher risk (HR 1.56, *p* = 0.002), while patients aged < 75 years with either early (HR 0.65, *p* < 0.001) or late referral (HR 0.62, *p* = 0.001) were at lower risk. Finally, patients aged over 75 years with late referral had more hospitalization days per year (coef 0.09, *p* < 0.001), while patients < 75 years with either early (coef − 0.07, *p* < 0.001) or late referral (coef − 0.05, *p* < 0.001) had fewer hospitalization days per year.

**Conclusions:**

Late referral of elderly patients prior to dialysis initiation is common and adversely associated with short- and long-term mortality as well as hospitalization days. Conversely, early referral of elderly patients is associated with a favorable short-term prognosis that is comparable to that of younger patients.

**Graphical abstract:**

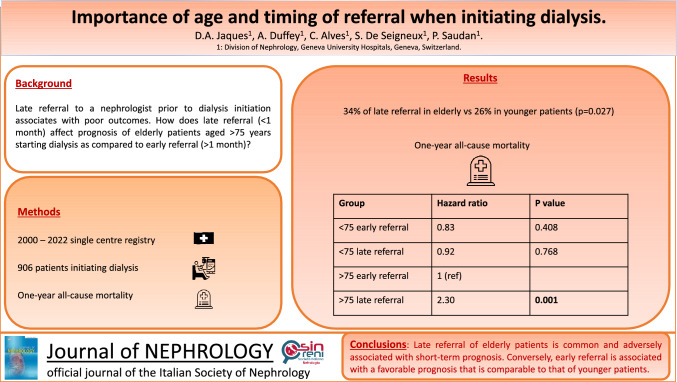

**Supplementary Information:**

The online version contains supplementary material available at 10.1007/s40620-025-02390-7.

## Introduction

Chronic kidney disease (CKD) affects more than 10% of the general population worldwide, with a total of over  800 million  affected individuals  [1]. Improvement of healthcare in the modern era has resulted in an increase in overall life expectancy. This however poses a significant challenge in dealing with age-related disease. Specifically, the elderly now constitute a growing population suffering from kidney failure in Western countries. According to North American data, the incidence of kidney failure in individuals over 75 years of age reached 1447 per million population in 2020, around three times the incidence of the 45- to 64-year-old age group [2]. Consequently, the elderly represent the fastest growing group of patients reaching kidney failure, with prevalence tripling from 7 to 20% in the past 30 years [3, 4]. As a result, nephrologists face an increasing number of requests to manage individuals on kidney replacement therapy (KRT). However, initiating dialysis in this population represents a significant challenge due to the multiple comorbidities, the inherent complications associated with the treatment itself, as well as its impact on the patients’ quality of life [5, 6]. Most importantly, the one-year mortality rate in patients over 75 initiating dialysis is notably high, exceeding 50% in several studies from the United States [7, 8]. In this setting, it has also been observed that the survival advantage conferred by KRT was likely minimal when compared to supportive care [9–13].

Late referral to the nephrologist prior to dialysis initiation is a factor associated with poor outcomes in patients suffering from kidney failure, manifesting with increased complications, more hospitalization days, and higher mortality rates [14, 15]. This is of particular concern in older individuals as late referral concerns as many as 60% of the patients included in prior studies [16, 17]. Altogether, the benefits of initiating dialysis could thus be even less evident in aging patients without proper referral to a nephrologist in a dedicated and timely manner. Specifically, whether there exists a combined effect of age and timing of referral to specialized care prior to KRT initiation on prognosis is currently unknown. In the present observational study, we investigated whether early referral (> 1 month prior to KRT initiation) to a nephrologist was associated with improved one-year survival in patients > 75 initiating KRT. Associations with overall mortality during follow-up and number of one-year hospitalization days represented secondary objectives.

## Methods

### Participants and setting

We conducted a retrospective analysis of a prospective cohort in a single university center (Geneva University Hospitals, Geneva, Switzerland). The Swiss renal registry and quality assessment program was established in 2006 by the Swiss Society of Nephrology on a voluntary basis and has been a legal obligation since 2013. Therefore, all patients in Switzerland on chronic dialysis are included in this registry and provide informed consent for their anonymized data to be used for quality control and clinical research purposes. In the present study, we included all adults initiating dialysis (incident patients) at our center from January 2000 to December 2022 for whom follow-up data to December 2023 were available. Patients already on dialysis (prevalent patients) or those requiring dialysis for acute kidney injury (functional alteration in kidney function lasting less than 3 months) were excluded. Demographic, clinical and laboratory data were retrieved from electronic medical records. Estimated glomerular filtration rate (eGFR) at KRT initiation was calculated with the 2012 Chronic Kidney Disease Epidemiology Collaboration (CKD-EPI) equation using serum creatinine measured at the very beginning of the first dialysis session.

### Dialysis treatment

Every incident kidney failure patient at our center is offered a pre-dialysis educational program regarding KRT modality (hemodialysis [HD], peritoneal dialysis [PD] or transplantation). Initial KRT modality is then decided with a strong emphasis on patient preference. Patients were dialyzed using either HD or hemodiafiltration (HDF) with post-dilution reinjection, according to the f attending physician’s decision. High flux polysulfone dialyzers were used with Braun (Braun, Melsungen, Germany) or Fresenius (Fresenius AG, Bad Homberg, Germany) dialysis machines. Patients choosing PD started with a continuous ambulatory PD (CAPD) incremental prescription, typically consisting of two glucose dwells during the day as well as one icodextrin dwell overnight. Prescription was then tailored according to the individual patient’s needs. Timing of referral was defined as the time between a first consultation with a nephrologist and initiation of dialysis. Early referral was defined as at least one consult with a nephrologist more than a month prior to dialysis initiation. Conversely, late referral was defined as the absence of such a consult in this timeframe. This one-month cut-off to define early referral/late referral was a priori decided based on previous evidence in a comparable setting in the Dialysis Outcomes and Practice Patterns Study (DOPPS) [14]. Also, on a local clinical basis, a one-month timeframe would typically allow for patients to initiate dialysis on a “permanent” access (i.e. tunneled cuffed or peritoneal catheter) rather than on a temporary catheter.

### Statistical analysis

Continuous variables are expressed as mean ± standard deviation (SD) and categorical ones as number and relative frequencies (%). Between-group comparisons were conducted using ANOVA and Chi-square for continuous and categorical variables, respectively. Primary outcome was one-year all-cause mortality according to age at dialysis initiation (< 75 or > 75) and timing or referral (early referral > 1 month or late referral < 1 month). Secondary outcomes were all-cause mortality over the entire follow-up as well as number of hospitalization days per year (i.e. during the first year of follow-up). Kaplan–Meier curves and log rank tests were performed to compare survival. Cox proportional hazard models were used with the patient group as a four-level categorical variable. Patients transplanted during follow-up, as well as those lost to follow-up were censored. Multivariate analyses included the following variables as potential confounders selected a priori: gender, Charlson Comorbidity Index, and dialysis modality (HD on catheter, HD on arteriovenous fistula or PD). The Charlson Comorbidity Index was used according to its original description (i.e. without accounting for age) [18]. In sensitivity analysis, a Fine and Gray regression model was applied, with transplantation treated as a competing risk assuming informative censoring of transplanted patients [19].

## Results

From 2000 to 2022, 906 patients initiated dialysis at our institution. Mean age was 62.1 ± 16.3 with 585 (64%) men. Four groups of patients were identified based on age at dialysis initiation (< 75 vs > 75 years old) and timing of referral to a nephrologist prior to dialysis initiation (early referral > 1 month vs late referral < 1 month). According to these criteria, 484 (53%) patients started dialysis at age < 75 years with early referral, 176 (19%) at age < 75 years with late referral, 162 (17%) at age > 75 with early referral and 84 (9%) at age > 75 years with late referral. As compared to younger patients, late referral to dialysis initiation was more common in elderly patients, with prevalence of 26% and 34%, respectively (*p* = 0.027) (Supplementary Table S1). Baseline characteristics of patients according to the four groups are described in Table 1. As compared to younger patients, elderly patients were less frequently smokers, had a lower prevalence of glomerulonephritis as a cause of kidney failure, and had higher Charlson Comorbidity Index. As compared to patients with early referral, those with late referral had a lower prevalence of hypertension and a higher prevalence of HD on catheter as the initial dialysis modality. Baseline characteristics of patients according to age groups are provided in Supplementary Table S2.

**Table 1:** Baseline characteristics according to age group and timing of referral

	< 75 ER (*N* = 484)	< 75 LR (*N* = 176)	> 75 ER (*N* = 162)	> 75 LR (*N* = 84)	*p* value
Age (years)	56 ± 13	53 ± 15	80 ± 3	80 ± 3	** < 0.001**
Gender (male)	314 (64%)	106 (60%)	114 (70%)	51 (60%)	0.221
Smoker	146 (30%)	57 (32%)	26 (16%)	14 (16%)	**0.001**
DM	199 (41%)	53 (30%)	65 (40%)	36 (42%)	0.059
HTN	407 (84%)	131 (74%)	148 (91%)	71 (84%)	** < 0.001**
Kidney failure etiology					
DM and/or HTN	232 (48%)	78 (44%)	118 (72%)	58 (69%)	** < 0.001**
GN	94 (19%)	36 (20%)	9 (5%)	6 (7%)	
Other	157 (32%)	61 (34%)	35 (21%)	20 (23%)	
Charlson Comorbidity Index	4 (3 – 6)	5 (3 – 7)	5 (4 – 6)	6 (4 – 7)	** < 0.001**
Dialysis modality					
HD on catheter	328 (67%)	164 (93%)	128 (79%)	82 (97%)	** < 0.001**
HD on AVF	82 (16%)	10 (5%)	18 (11%)	2 (1%)	
PD	74 (15%)	2 (1%)	16 (9%)	0 (0%)	
eGFR (mL/min/1.73m^2^)	7 (5 – 9)	6 (4 – 8)	7 (5 – 9)	6 (5 – 8)	0.825

Bold values indicate *p* < 0.05

*ER* early referral; *LR* late referral; DM, diabetes mellitus; *HTN* hypertension; *GN* glomerulonephritis; *HD* hemodialysis; *AVF* arteriovenous fistula; *PD* peritoneal dialysis; *eGFR* estimated glomerular filtration rate As far as I can see, none of the changes I made to the tables in the original review were made here.

**Fig. 1:**
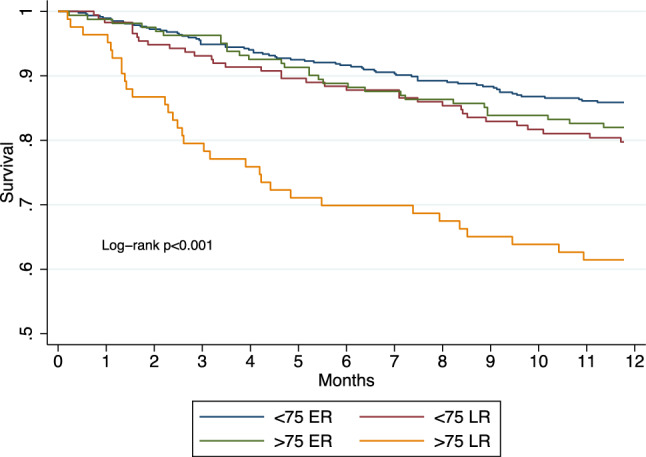
Kaplan–Meier survival function for one-year all-cause mortality according to patient group

**Fig. 2:**
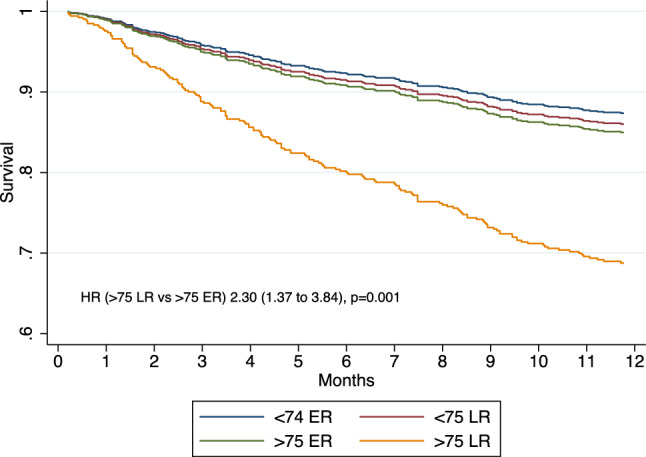
Multivariate Cox survival function for one-year all-cause mortality according to patients’ group. Multivariate model is adjusted for the following covariates: Gender, Charlson score and dialysis modality (HD on catheter, HD on AVF or PD)

**Fig. 3:**
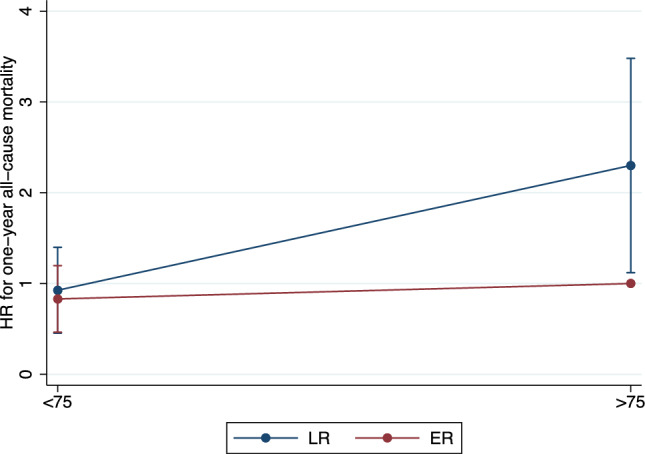
Hazard ratio estimates from multivariate Cox survival function for one-year all-cause mortality according to patients’ age and timing or referral. Multivariate model is adjusted for the following covariates: Gender, Charlson score and dialysis modality (HD on catheter, HD on AVF or PD). Patients > 75 ER are considered the reference category

Mean follow-up time was 39.4 ± 36.1 months during which 573 (64%) patients died and 256 (28%) underwent kidney transplant. During the first year after dialysis initiation, 160 (17%) patients died, corresponding to an all-cause mortality rate of 20.5% person-years. Kaplan–Meier survival function for one-year all-cause mortality according to patient group is illustrated in Fig. 1. Patients > 75 with late referral had increased mortality risk as compared to the three other groups (< 75 with ER, < 75 with late referral and > 75 with early referral) (*p* < 0.001 for log-rank test). In univariate Cox proportional hazard regression for one-year all-cause mortality, considering patients > 75 with early referral as the reference category, patients > 75 with late referral had increased mortality risk (Hazard Ratio [HR] 2.58, 95% CI 1.56 to 4.26, *p* < 0.001) while patients < 75 with either early (HR 0.77, 95%CI 0.49 to 1.19, *p* = 0.244) or late referral (HR 1.13, 95%CI 0.69 to 1.86, *p* = 0.611) had similar mortality risks. In multivariate Cox proportional hazard regression for one-year all-cause mortality, considering patients > 75 with early referral as the reference category, patients > 75 with late referral had increased mortality risk while patients < 75 with either early or late referral had similar mortality risks (Table 2). The interacting term “age X referral” was significant (*p* = 0.031), confirming a differential effect of referral depending on age. Among the considered covariates, the Charlson Comorbidity Index was positively associated with mortality. Moreover, considering HD on catheter as the reference modality, initiation with HD on arteriovenous fistula was negatively associated with mortality while initiation with PD was not. Finally, gender was not associated with mortality. Multivariate Cox survival function for one-year all-cause mortality according to patient group is illustrated in Fig. 2. Hazard ratio estimates for one-year all-cause mortality, considering patients > 75 with early referral as the reference category, are illustrated in Fig. 3.

**Table 2:** Associations with one-year all-cause mortality using multivariate Cox proportional hazard regression

	HR (95% CI)	*p* value
Group		
< 75 ER	0.83 (0.53 to 1.29)	0.408
< 75 LR	0.92 (0.55 to 1.54)	0.768
> 75 ER	1 (reference)	
> 75 LR	2.30 (1.37 to 3.84)	**0.001**
Gender (male)	0.85 (0.61 to 1.19)	0.365
Charlson Comorbidity Index	1.28 (1.21 to 1.36)	** < 0.001**
Dialysis modality		
HD on catheter	1 (reference)	
HD on AVF	0.36 (0.15 to 0.83)	**0.016**
PD	0.84 (0.45 to 1.55)	0.588

Bold values indicate *p* < 0.005

*ER* early referral; *LR* late referral; *HD* hemodialysis; see note in Table 1 ; *AVF* arteriovenous fistula; *PD* peritoneal dialysis

In the multivariable Cox proportional hazard regression for overall all-cause mortality over the entire follow-up, considering patients aged > 75 years with early referral as the reference category, patients > 75 years old with late referral had increased mortality risk (HR 1.56, 95% CI 1.18 to 2.07, *p* = 0.002) while patients < 75 years old with either early (HR 0.65, 95% CI 0.54 to 0.80, *p* < 0.001) or late referral (HR 0.62, 95% CI 0.47 to 0.81, *p* = 0.001) had lower mortality risks. Multivariate Cox survival function for overall all-cause mortality over the entire follow-up according to patient group is illustrated in Supplementary Fig. S1.

**Fig. 4:**
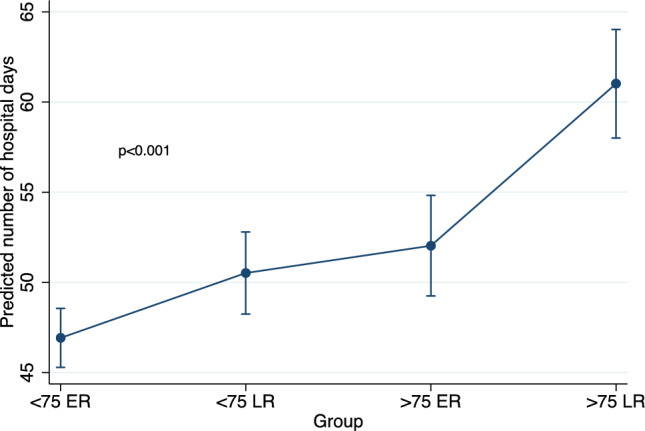
Predicted number of one-year hospitalization days according to patient groups using multivariate zero-inflated Poisson regression model. Multivariate model is adjusted for the following covariates: Gender, Charlson score and dialysis modality (HD on catheter, HD on AVF or PD)

Mean hospitalization days during the first year of dialysis were 25(8 – 56), 36(15 – 63), 36(9 – 74) and 48(21 – 84) for patients < 75 years old with early referral, < 75 years old with late referral, > 75 years old with early referral and > 75 years old with late referral, respectively. In a multivariate zero-inflated Poisson regression model, considering patients > 75 with early referral as the reference category, patients > 75 years old with late referral had higher predicted one-year hospitalization days, while patients < 75 years old with either early or late referral had lower predicted one-year hospitalization days (Table 3). Among considered covariates, male gender was associated with lower predicted one-year hospitalization days, while Charlson Comorbidity Index was associated with higher predicted one-year hospitalization days. Finally, considering HD on catheter as the reference modality, initiation with HD on arteriovenous fistula or PD was associated with lower predicted one-year hospitalization days. Predicted number of one-year hospitalization days according to patient group using a multivariate zero-inflated Poisson regression model is illustrated in Fig. 4.

**Table 3:** Associations with one-year hospitalization days using multivariate zero-inflated Poisson regression model

	Coeff (95% CI)	*p* value
Group		
< 75 ER	– 0.07 (– 0.10 to – 0.05)	** < 0.001**
< 75 LR	– 0.05 (– 0.08 to – 0.02)	** < 0.001**
> 75 ER	0 (reference)	
> 75 LR	0.09 (0.06 to 0.13)	** < 0.001**
Gender (male)	– 0.09 (– 0.11 to – 0.07)	** < 0.001**
Charlson Comorbidity Index	0.05 (0.05 to 0.06)	** < 0.001**
Dialysis modality		
HD on catheter	0 (reference)	
HD on AVF	– 0.40 (– 0.43 to – 0.36)	** < 0.001**
PD	– 0.47 (– 0.51 to – 0.43)	** < 0.001**

Bold values indicate* p* < 0.005

*ER* early referral; *LR* late referral; *HD* hemodialysis; see note in Table 1; *AVF* arteriovenous fistula; *PD* peritoneal dialysis

In the sensitivity analysis, a Fine and Gray regression model with transplantation treated as a competing risk for one-year all-cause mortality was used. Results (Supplementary Table S3) were qualitatively identical to the main model.

## Discussion

In this observational study, we found that late referral (< 1 month) of elderly patients (> 75years old ) to a nephrologist prior to dialysis initiation is common and associated with a substantial increase in mortality during the first year of dialysis. Late referral is also adversely associated with overall survival as well as hospitalization days in the first year after dialysis start in this elderly population. Conversely, early referral (> 1 month) of elderly patients is associated with a favorable short-term prognosis that is comparable to that of younger patients.

With the increasing number of elderly patients reaching kidney failure, the debate among nephrologists regarding the provision of KRT has been prominent over the past years based on the perceived dim prognosis in this specific population. North American data on patients > 75 initiating dialysis reported a one-year mortality rate reaching 45% to 55% [7, 8]. In Europe, observational studies in elderly patients from the UK and the Netherlands indicated that the survival advantage conferred by KRT as compared to conservative care is minimal, particularly in highly comorbid patients [9, 10]. Moreover, recent observations in Canadian patients highlighted that elderly patients receiving maintenance dialysis spent more time in hospital and were more commonly admitted to the intensive care unit as compared to those opting for conservative care [11, 12]. Finally, in a recent target trial emulation study including more than 20,000 kidney failure participants over 65 of age, patients initiating KRT had marginally longer survival but spent more days in the hospital as compared to those entirely foregoing dialysis [13]. Besides the decision to initiate dialysis itself, late referral to nephrology care prior to dialysis start is of particular concern in the elderly population [14, 15]. There is unfortunately no uniform definition of late referral in this setting, with timing broadly spanning from 1 to 6 months [20]. Regardless, late referral prior to dialysis is overall frequent in the elderly. In the French REIN registry capturing more than 24,000 patients over the age of 75 initiating dialysis between 2005 and 2012, 31% of patients started KRT on an emergency basis under life-threatening circumstances [16]. In a German study on 254 patients initiating HD between 1998 and 2001 at a single center, late referral (< 2 months) concerned 42% of younger patients but as many as 60% of patients above 75 years old [17]. In the present study, even opting for a more stringent cut-off (< 1 month), we confirmed those findings and observed that late referral was more common in elderly patients (34%) as compared to younger patients (26%). A first consultation  with a nephrologist at least in the months preceding dialysis is of prime importance as late referral has consistently been associated with poor prognosis. In a French cohort of 107 octogenarian patients initiating dialysis, late referral (< 4 months) was the strongest predictor of early mortality [21]. Similarly, in 84 incident elderly patients > 75 years old , unplanned initiation of dialysis significantly impacted mortality, with one-year survival of 14% vs 73% for early vs late referral (< 3 months), respectively [22]. These data collectively raise the question of the possible interaction between advanced age and timing of referral prior to dialysis initiation on the overall prognosis of those patients. In an important publication, Schwenger et al. reported the outcomes of 254 patients initiating HD between 1998 and 2001 in a single center in Germany [17]. While late referral (< 2 months) was independently associated with poor one-year survival, the relative risk of death conferred by late referral was similar in elderly or younger patients. In this regard, the Authors could not show an interaction between age and timing of referral on short-term prognosis. This is in marked contrast with the findings of our present study, where late referral was strongly associated with one-year mortality in elderly patients, but not in younger ones. Conversely, we found that early referral in elderly patients was associated with a favorable short-term prognosis, comparable to that of younger patients. Contrary to Schwenger et al., we thus found that the impact of late referral on short-term prognosis was radically different depending on age, as elderly patients suffer greatly from late referral prior to dialysis initiation while younger patients seem relatively unaffected. This effect was observed not only for one-year mortality but also regarding one-year hospitalization days as well as overall mortality during follow-up. Among the notable differences with the prior study by our German colleagues, we included a much larger sample size with a higher level of statistical adjustment. More importantly, these two studies were conducted more than two decades apart, potentially implying important differences in the routine care of dialysis patients, in particular in the aging population. Finally, we used a more stringent definition for late referral with the absence of a nephrology consult within a month prior to dialysis initiation. As no uniform definition of late referral exists in this setting, various cut-offs have been used resulting in different degrees of prevalence of patients fulfilling those criteria with potentially different clinical implications [20]. In this regard, our results show that a single contact with a nephrologist at least one month prior to dialysis initiation is associated with a markedly improved short-term prognosis in the elderly population, that is thus comparable to that of younger patients. The clinical relevance of this finding is highlighted by the particularly high mortality rates observed in elderly patients during the first months of dialysis treatment [23].

As expected, the proportion of patients initiating HD on a catheter in our study was substantially higher in those with late referral as compared to early referral to dialysis. Furthermore, in the multivariable analysis, we confirmed that initiation of dialysis on a catheter, as opposed to a fistula, was independently associated with higher mortality, regardless of the timing or referral to dialysis. Consequently, our results highlight that late referral directly impacts the patient’s prognosis beyond what could be inferred from the initial type of vascular access alone. This potentially reflects the numerous and metabolic imbalances of the late-referred patient such as anemia, malnutrition, phosphocalcic abnormalities, hypertension and fluid overload, all of which have been linked to poor outcomes [24].

Readers must bear in mind certain limitations of our study. Most important is the observational nature of the study, limiting causative inference of the findings. Specifically, late referral to dialysis might represent a marker of poor prognosis rather than a direct causal factor. It must however be pointed out that late referral was consistently associated with poor outcome despite adjustment for comorbidity level and type of vascular access. Beyond these technical considerations, our results provide relevant clinical information in that they illustrate the importance of early dialysis planning overall in the growing elderly population. The second significant limitation of our study is the static definition of a one-month timeframe to define early and late referral. As this variable was prospectively captured in our database as a single binary variable, a posteriori analysis of longer timeframes of referral was unfortunately limited. However, our results suggest that a one-month referral period is sufficient to favorably modify the prognosis of elderly patients initiating dialysis. Finally, our study is single-center in design, rendering extrapolation of our findings to other settings difficult. In particular, our conclusions would not directly apply to low and middle-income countries where resources dedicated to the care of elderly patients suffering from kidney failure are limited.

Using a single-center prospective registry spanning the last two decades in Switzerland, we confirm that late referral to dialysis is frequent in the elderly population. We found that late referral is associated with poor short- and long-term prognosis in elderly patients, with a substantial increase in mortality as well as hospitalization days. Conversely however, early referral to dialysis is associated with much more favorable outcomes, that are comparable to those of younger patients. These findings highlight the importance of an overall plan of care in the setting of kidney failure management in the aging population and could inform clinical decisions when individually deciding on the relevance of KRT initiation.

## Supplementary Information

Below is the link to the electronic supplementary material.

Supplementary file1 (DOCX 89 KB)

## Data Availability

The data supporting the findings of this study are available from the corresponding author upon reasonable request.
